# Strategy for Co‐Enhancement of Remanence–Coercivity of RE‐Fe‐B Sintered Magnets with High Ce‐Content: Appropriate La Substitution

**DOI:** 10.1002/advs.202301312

**Published:** 2023-04-17

**Authors:** Hao Chen, Yuqing Li, Hongguo Zhang, Weiqiang Liu, Haihui Wu, Yuan Qin, Ming Yue, Qifeng Wei, Baoguo Zhang, Jinghui Di

**Affiliations:** ^1^ Faculty of Materials and Manufacturing Key Laboratory of Advanced Functional Materials Ministry of Education of China Beijing University of Technology Beijing 100124 China; ^2^ Hangzhou Foresee Technology Co., Ltd Hangzhou 311500 China; ^3^ Hangzhou Magmax Technology Co., Ltd Hangzhou 311500 China

**Keywords:** Ce‐valence, grain boundaries, high‐abundance La/Ce, magnetic properties, REFe_2_ phases

## Abstract

The development of low‐cost RE‐Fe‐B sintered magnets with large La/Ce content is of great significance for the balanced utilization of rare earth (RE) resources, but it is limited by reduced magnetic properties. In this work, the coercivity (*H*_cj_), remanence (*B*_r_), maximum energy product [(*BH*)_max_], and temperature stability are simultaneously enhanced for magnets with LaCe accounting for 40 wt% of the total RE. The synergistic regulation of the REFe_2_ phase, Ce‐valence, and grain boundaries (GBs) in RE‐Fe‐B sintered magnets is realized for the first time by introducing appropriate La elements. The La elements inhibit the generation of the REFe_2_ phase and tend to stay in the triple junctions, promoting the segregation of the RE/Cu/Ga elements and contributing to the formation of Ce/Nd/Cu/Ga‐rich continuous thicker lamellar GBs, and as a result, weakening the detrimental effect on HA caused by La element substitution and enhancing *H*_cj_. In addition, partial La atoms entering the RE_2_Fe_14_B phase are beneficial for improving the *B*_r_ and temperature stability of the magnets and promoting the Ce^3+^ ion ratio, which also provides additional benefit for *B*_r_. The findings provide an effective and feasible way to co‐enhance the remanence and coercivity of RE‐Fe‐B sintered magnets with high Ce content.

## Graphical abstract

A novel strategy of appropriate La substitution in Ce‐containing sintered RE‐Fe‐B magnet to overcome remanence–coercivity trade‐off is described. The comprehensive optimization of microstructure, including the REFe_2_ phase, Ce‐valence in main phase, and grain boundaries is realized, which overcomes the contradiction brought by the intrinsic magnetic properties, and achieves the comprehensive improvement of remanence, coercivity, maximum energy product, and temperature stability.

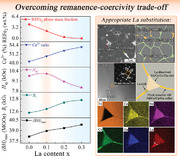

## 1 Introduction

In recent years, the concept of sustainable development has attracted widespread attention in the new energy technology industry, leading to an enormous demand for permanent magnets.^[^ ^1^, ^2^, ^3^ ^]^ High‐performance Nd‐Fe‐B sintered permanent magnets play a crucial role in many energy‐saving technologies such as wind turbines and new energy vehicles.^[^ ^4^, ^5^ ^]^ Unfortunately, the prices of rare earth (RE) elements Pr/Nd, as nonrenewable resources, have increased significantly, due to high demand. Critically, this has created a long‐term backlog of enormous quantities of inexpensive and abundant La/Ce, which can be detrimental to the efficient utilization of RE resources.^[^ ^6^, ^7^ ^]^ In addition, compared to Ce, the utilization of La elements in permanent magnets is restricted to a lower level due to the extreme worsening of *H*_cj_.^[^ ^8^, ^9^, ^10^ ^]^ Therefore, the development of RE‐Fe‐B sintered magnets based on high‐abundance RE elements (La and Ce) is of great strategic significance, in terms of economic efficiency and sustainable development. However, high Ce‐content substitution in sintered Nd‐Fe‐B magnets will lead to reduced *B*_r_ and *H*_cj_, due to the lower intrinsic magnetic properties of Ce_2_Fe_14_B [saturation magnetization (*M*_s_) of 11.7 kG, magnetocrystalline anisotropy field (*H*_A_) of 26 kOe].^[^ ^11^, ^12^, ^13^, ^14^ ^]^ Compared to Ce_2_Fe_14_B, La_2_Fe_14_B has a higher *M*_s_ (13.8 kG), but lower *H*_A_ (20 kOe).^[^ ^11^ ^]^ In terms of intrinsic magnetic properties, the substitution of La for Ce in sintered magnets may increase *B*_r_ and decrease *H*_cj_, resulting in a remanence–coercivity trade‐off. However, this remanence–coercivity trade‐off exists in all permanent magnetic materials. Composition and structure design may provide new opportunities for overcoming the trade‐off between remanence and coercivity.^[^ ^15^ ^]^ For example, excellent *B*_r_ and *H*_cj_ combinations have been obtained in nanocomposite permanent magnets through the design of heterostructures, and record‐high energy product has been obtained.^[^ ^1^ ^]^ Consequently, simultaneously improving *B*_r_ and *H*_cj_ with LaCe‐contained sintered magnets has become a significant scientific issue and fundamental challenge for improving magnetic properties.

In addition to the intrinsic magnetic properties, the substitution of La for Ce in RE‐Fe‐B sintered magnets can also lead to a series of changes in the magnet, with a non‐negligible effect on the magnetic properties. Interestingly, the La element will enter the CeFe_2_ phase, reducing lattice stability and finally leading to decomposition.^[^ ^16^ ^]^ Recent studies have demonstrated that when the Ce content in Nd‐Ce‐Fe‐B sintered magnets exceeds a certain amount, the CeFe_2_ phase will inevitably develop.^[^ ^17^, ^18^, ^19^, ^20^ ^]^ For example, the mass fraction of the CeFe_2_ phase in a magnet was found to reach 4.94 wt% when Ce represented 50 wt% of the total RE, whereas the mass fraction of the other RE‐rich phase was only 3.14 wt%.^[^ ^20^ ^]^ Because the CeFe_2_ phase will be paramagnetic at room temperature, the appearance of the CeFe_2_ phase in sintered magnets will unavoidably decrease the *B*_r_.^[^ ^21^, ^22^ ^]^ Meanwhile, the production of the CeFe_2_ phase will consume Ce and Fe elements, leading to a reduction of the RE_2_Fe_14_B phase in the magnet, which is also detrimental to magnetic properties.^[^ ^16^, ^23^, ^24^ ^]^ In addition, the paramagnetic CeFe_2_ phase with large block aggregation distribution can increase the local demagnetization field, leading to a reduction of the nucleation field. Unfortunately, CeFe_2_ phases also occupy considerable amount of Fe, which will increase the Fe content of lamellar grain boundary (GB) decreasing the effect of suppressing magnetic coupling between grains and leading to a significant decrease in *H*_cj_.^[^ ^25^, ^26^ ^]^ By contrast, it has also been claimed that the CeFe_2_ phase can contribute to the process of sintering because of its low melting point (925 °C).^[^ ^17^, ^19^ ^]^ During sintering, the melted CeFe_2_ phase will increase the volume fraction of the liquid phase and promote the formation of continuous lamellar GBs, to isolate adjacent ferromagnetic grains.^[^ ^17^ ^]^ Therefore, it is essential to clarify how the CeFe_2_ phase affects *H*_cj_, to control the microstructure and enhance the magnetic properties. Undoubtedly, the elimination of the CeFe_2_ phase by the La element should result in subversive alterations to the morphology, structure, and composition of the GB phases in sintered magnets. As a result, replacing a portion of Ce with La may provide an opportunity to regulate the CeFe_2_ and GB phases, which is one of the keys to obtaining high *H*_cj_ of LaCe‐based RE‐Fe‐B sintered magnets.

Furthermore, the La element has shown a regulatory effect on Ce‐valence in the Ce_2_Fe_14_B phase, which is also an influential factor for Ce‐contained sintered RE‐Fe‐B magnets. By contrast to Pr/Nd/La elements with a stable +3 valence, the Ce element in the RE‐Fe‐B alloy has regulatable +3 and +4 mixed valence, but the +4 valence is not magnetic. Stabilizing the Ce^3+^ configuration with a local 4f moment can increase the intrinsic magnetic properties;^[^ ^27^, ^28^, ^29^ ^]^ thus, the magnetic properties of the Nd‐Ce‐Fe‐B magnet will be influenced by the Ce‐valence. Notably, the steric environment of Ce atoms and the Ce‐valence are closely related, with the Ce‐valence gradually changing to +3 valence as the lattice volume increases.^[^ ^30^, ^31^, ^32^ ^]^ Based on the density functional theory, Alam et al.^[^ ^30^ ^]^ demonstrated that La‐doping facilitated the transfer of the Ce‐valence to Ce^3+^. Jin et al.^[^ ^31^ ^]^ found that the entry of large radius La^3+^ into the 2:14:1 phase lattice caused the Ce‐valence to migrate toward Ce^3+^, with favorable magnetic characteristics in the magnets prepared by co‐doping the La_35_Ce_65_ alloy. Therefore, it may be beneficial to further enhance magnetic properties by replacing Ce with large radius La to induce the conversion of the Ce valence to Ce^3+^. Controlling the substitution amount of the La element may provide new opportunities for breaking the trade‐off between remanence and coercivity. The substitution of La for Ce can increase *M*_s_ and promote the valence migration of Ce to the +3 valence with favorable magnetic properties, which may enhance the *B*_r_ of sintered magnets. In addition, the suppression of the REFe_2_ phase in the magnet by La instead of Ce will provide an opportunity for regulating the GB phase structure to improve *H*_cj_.

In this work, we realized the synergistic regulation of the ReFe_2_ phase content, Ce‐valence, and GB phase structure for the first time in single‐main‐phase [(La*_x_* Ce_1−_ *_x_*)_0.4_Nd_0.4_Pr_0.2_]_31_Fe_bal_M_1.95_B_0.96_ (*x* = 0, 0.1, 0.2, and 0.3) sintered magnets by using the La element to design the composition. More importantly, in addition to *B*_r_ improvement, enhancement was realized, rather than an expected reduction in *H*_cj_, breaking the remanence–coercivity trade‐off. By investigating the phase composition, Ce‐valence, microstructure characteristics, and magnetization reversal behavior in the magnets, we revealed the influence mechanism of the changes in magnetic properties after La replaced Ce and the origin of simultaneous improvement in remanence–coercivity.

## 2 Results

### 2.1 Magnetic Properties

**Figure 1:**
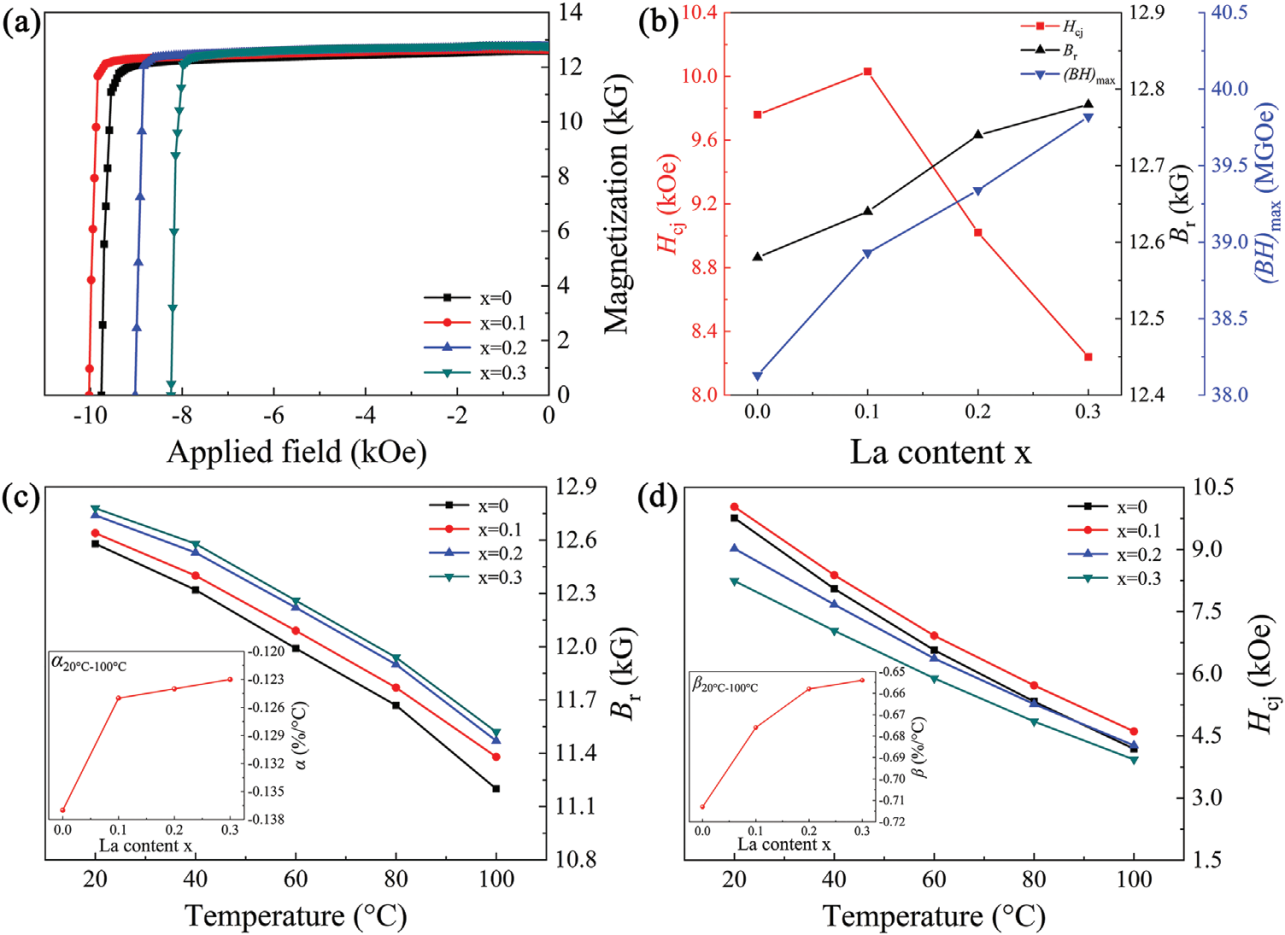
Magnetic characteristics of the (La*_x_* Ce_1−_ *_x_*)‐40 (*x* = 0–0.3) magnets: a) demagnetization curves at room temperature for all magnets; b) variations in the magnetic properties with La content *x*; c,d) variations in *B*_r_ and *H*_cj_ of the four magnets with the test temperatures (the insets show the *α* and *β* in the temperature range of 20–100 °C as a function of La substitution).

To determine the optimal magnetic properties of the samples with different La doping amounts, we systematically investigated the correlation between the manufacturing process and the magnetic properties of the (La*_x_* Ce_1−_ *_x_*)‐40 (*x* = 0–0.3) magnets. The optimal sintering and annealing process was determined from the room temperature (20 °C) demagnetization curves of the magnets sintered and annealed at different temperatures (Figures S1–S3, Supporting Information) and the magnetic property data (Tables S1–S3, Supporting Information). **Figure** **1**a,b shows the room temperature demagnetization curves and the magnetic properties of the magnets with different La substitutions (*x*) under the optimal sintering and annealing process. The *H*_cj_, *B*_r_, and (*BH*)_max_ values of the *x* = 0.1 magnet increased to 10.03 kOe, 12.64 kG, and 38.93 MGOe, respectively, compared to the *x* = 0 magnet with corresponding values of 9.76 kOe, 12.58 kG, and 38.13 MGOe, respectively. However, as *x* continuously increased, the *H*_cj_ gradually declined. As a result, the *H*_cj_ of the magnets with *x* = 0.2 and 0.3 decreased to 9.02 kOe and 8.24 kOe, respectively. Unlike the *H*_cj_ variations, the *B*_r_ and (*BH*)_max_ values of the magnets with *x* = 0.2 and 0.3, which were 12.74 kG, 39.34 MGOe and 12.78 kG, 39.82 MGOe, respectively, monotonically increased with increasing La substitution. Compared to Ce_2_Fe_14_B, which had *M*_s_ and *H*_A_ values of 11.7 kG and 26 kOe, respectively, La_2_Fe_14_B had a higher *M*_s_ (13.8 kG) and a lower *H*_A_ (20 kOe) value.^[^ ^11^ ^]^ Therefore, in terms of the intrinsic magnetic properties, the substitution of La for Ce would inevitably lead to a decrease in the *H*_cj_ and an increase in *B*_r_. Surprisingly, in the magnet with *x* = 0.1, the *H*_cj_, *B*_r_, and (*BH*)_max_ values simultaneously improved. This meant that the change in magnetic properties after La replaced Ce was not only controlled by the intrinsic magnetic properties but was also dependent on the phase composition and microstructure characteristics.

Another critical parameter, also a key parameter for the application of magnets, was temperature stability. According to the demagnetization curves and magnetic properties of the magnets at different temperatures (Figure S4 and Table S4, Supporting Information), the temperature stability of the magnets was also evaluated by La substitution. The temperature‐dependent *B*_r_ and *H*_cj_ of the magnets with *x* values from 0 to 0.3 are shown in Figure 1c,d. In the temperature range of 20–100 °C, the *B*_r_ of the magnets increased with increasing La substitution, and the remanence coefficient *α* gradually improved from −0.137% °C^−1^ for *x* = 0 to −0.123% °C^−1^ for *x* = 0.3 (Figure 1c). As shown in Figure 1d, the *H*_cj_ of the magnet with *x* = 0 decreased the fastest with increasing temperature, and its *H*_cj_ (4.19 kOe) at 100 °C was even lower than that of the magnet with *x* = 0.2 (4.27 kOe). Furthermore, the magnet with *x* = 0.1 had the highest *H*_cj_ at all test temperatures. Consistent with the trend of *α*, the coercivity coefficient *β* also gradually improved from −0.713% °C^−1^ for *x* = 0 to −0.654% °C^−1^ for *x* = 0.3. In addition, the *α* and *β* values of the magnets in all temperature intervals were enhanced with increasing La substitution (Table S4, Supporting Information). The above results showed that La substitution for Ce significantly improved the temperature stability of the magnets. This was because the *T*_c_ of La_2_Fe_14_B (257 °C) was much higher than that of Ce_2_Fe_14_B (151 °C), and the La_2_Fe_14_B phase showed the weak temperature dependence of *H*_A_.^[^ ^11^, ^33^ ^]^

### 2.2 Phase Constitution, Ce‐Valence, and Microstructure

**Figure 2:**
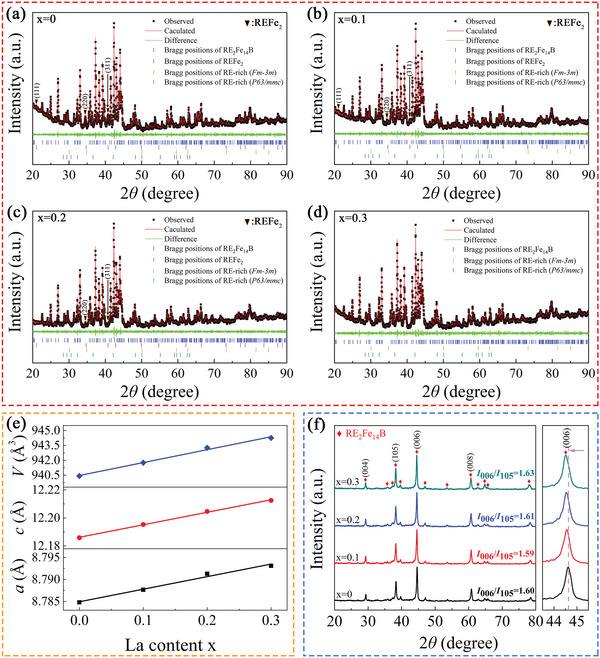
a–d) XRD refinement results of the (La*_x_* Ce_1−_ *_x_*)‐40 (*x* = 0–0.3) magnets, where the observed and calculated patterns are shown in black and red colors, respectively, while the differences are shown in green; e) variations of the 2:14:1 phase lattice parameter with the amount of La substitution *x*; f) XRD patterns of the four magnets evaluated along the alignment plane.

X‐ray diffraction (XRD) Rietveld refinement was used to investigate the phase compositions of the (La*_x_* Ce_1−_ *_x_*)‐40 (*x* = 0–0.3) magnets, as shown in **Figure** **2**a–d. The four magnets were dominated by the RE_2_Fe_14_B tetragonal phase (space group *P4_2_/mnm*) with a trace amount of the REFe_2_ phase (space group *Fd* $\bar{3}$ *m*) and metallic RE‐rich phase (RE = Pr/Nd/Ce/La). Compared to the RE_2_Fe_14_B tetragonal phase, REFe_2_ also belonged to the intergranular RE‐rich phase. The following RE‐rich phase exclusively referred to the traditional metallic RE‐rich phase, excluding the REFe_2_ phase, because the REFe_2_ phase and the traditional metallic RE‐rich phase had different effects on the magnetic properties and microstructure. As shown in Table 1, the mass fraction of the RE_2_Fe_14_B phase steadily increased with increasing La substitution, from 94.45 wt% for *x* = 0 to 96.50 wt% for *x* = 0.3. By contrast, the mass fraction of the REFe_2_ phase in the magnets gradually decreased from 4.07 wt% for *x* = 0 to 0.38 wt% for *x* = 0.2, and the REFe_2_ phase was not detected in the magnet for *x* = 0.3. This also proved that the substitution of La could inhibit the formation of the REFe_2_ phase, which was consistent with previous reports.^[^ ^16^, ^34^, ^35^ ^]^ Because the presence of the REFe_2_ phase consumed some of the Ce and Fe elements, a decrease in the REFe_2_ phase content promoted an increase in the RE_2_Fe_14_B phase content. However, remanence in the Nd‐Fe‐B sintered magnets was influenced by the *c*‐axis alignment degree, density, and mass fraction of the main phase.^[^ ^36^ ^]^ The gradual increase of the RE_2_Fe_14_B phase mass fraction in the magnets with increasing La substitution was also one of the reasons for the increase in *B*_r_. In addition, the lattice parameters *a*, *c*, and *V* of the RE_2_Fe_14_B phase gradually increased as La substitution increased (Figure 2e), indicating that the La atoms entered the RE_2_Fe_14_B phase lattice in all magnets. Evidently, this would lead to an increase in *M*_s_ and a decrease in *H*_A_ for the RE_2_Fe_14_B phase. In terms of the intrinsic magnetic properties, it was beneficial to improve the *B*_r_ of the sintered magnets, but this would lead to a deterioration of *H*_cj_. Moreover, the decrease in the REFe_2_ phase mass fraction also led to an increase and composition change of the RE‐rich phase, which was beneficial to the *H*_cj_ of the magnets. The four magnets had two different types of RE‐rich phases, namely, type I RE‐rich phase with a cubic structure (space group *Fm‐3m*) and type II RE‐rich phase with a hexagonal structure (space group *P63/mmc*). These were also the two types of RE‐rich phases commonly observed in RE‐Fe‐B sintered magnets reported in the literature.^[^ ^17^, ^37^, ^38^ ^]^ Previous studies demonstrated that the RE‐rich phase with a cubic structure in the triple junctions could reduce lattice distortion at the interface, along with better wettability of the RE_2_Fe_14_B grains, which was conducive to the formation of continuous lamellar GBs.^[^ ^38^, ^39^, ^40^ ^]^ Contrarily, higher lattice misfit was observed between the RE_2_Fe_14_B phase and the RE‐rich phase with a hexagonal structure at the triple junctions, which was detrimental to *H*_cj_.^[^ ^38^ ^]^ In the magnets with *x* = 0, the mass fractions of the type I RE‐rich phase and type II RE‐rich phase were 0.31 and 1.17 wt%, respectively. With an increase in La substitution amount, the mass fraction of the type I RE‐rich phase initially increased and then decreased, peaking at 1.34 wt% when *x* = 0.1. By contrast, the type II RE‐rich phase increased monotonously with an increase in x, and the mass fraction in the *x* = 0.3 magnet reached 2.69 wt%. The XRD results demonstrated that La substitution decreased the mass fraction of the REFe_2_ phase and changed the RE‐rich phase composition in the magnets. Of note, the decrease in mass fraction of the REFe_2_ phase produced additional Ce elements, which could form more type I RE‐rich phases. However, excessive La substitution (*x* ≥ 0.2) led to a decrease in Ce element content, which decreased the mass fraction of the type I RE‐rich phase, while the mass fraction of the type II RE‐rich phase rapidly increased. Later, we will reveal the reason for the change in RE‐rich phase composition and the influence mechanism on the coercivity by analyzing the microstructure. The XRD patterns of the four magnets tested by the surface perpendicular to the easy axis are shown in Figure 2f. Unlike the powder XRD patterns, the intensities of the (004), (105), (006), and (008) diffraction peaks of the RE_2_Fe_14_B phase were sharply enhanced. The intensity ratio, *I*_(006)_/*I*_(105)_, has typically been used to characterize the alignment degree in the Nd‐Fe‐B sintered magnets.^[^ ^41^ ^]^ The *I*_(006)_/*I*_(105)_ values of the four magnets were essentially identical, ≈1.6, suggesting that the alignment degree was almost the same. This indicated that the variations in *B*_r_ with an increase in La substitution were unaffected by the alignment fluctuations. In addition, the (006) diffraction peak also moved to the left, from 44.62° (*x* = 0) to 44.52° (*x* = 0.3), providing more evidence of the lattice volume expansion caused by the entrance of La with a larger atomic radius into the 2:14:1 phase.

**Table 1:** Mass fractions of the different phases evaluated by Rietveld analysis and the *R* factors for the (La*_x_* Ce_1−_ *_x_*)‐40 (*x* = 0–0.3) magnets

	Mass fraction [wt%]				*R* factors		
La content (*x*)	RE_2_Fe_14_B (*P4_2_/mnm*)	REFe_2_ *Fd* $\bar{3}$ *m*	RE‐rich (*Fm‐3m*)	RE‐rich (*P63/mmc*)	*R*_p_	*R*_wp_	*R*_exp_
0	94.45	4.07	0.31	1.17	1.94	2.34	2.04
0.1	95.16	2.13	1.34	1.37	2.43	2.81	2.54
0.2	96.46	0.38	1.22	1.94	2.57	2.93	2.66
0.3	96.50	–	0.81	2.69	2.33	2.66	2.36

**Figure 3:**
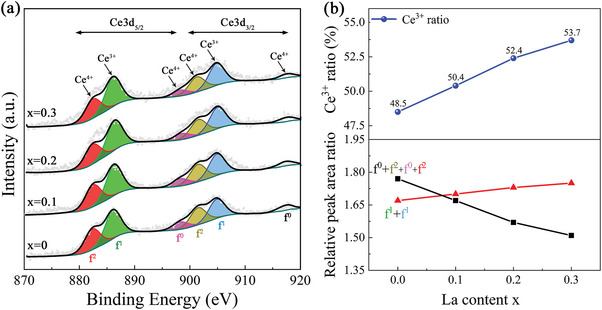
a) XPS spectra of the Ce 3d level in the (La*_x_* Ce_1−_ *_x_*)‐40 (*x* = 0–0.3) magnets; b) relative area ratio of the Ce^3+^ and Ce^4+^ peaks and the variations in the Ce^3+^ ratio with La substitution amount *x*.

The variations in Ce valence in the (La*_x_* Ce_1−_ *_x_*)‐40 (*x* = 0–0.3) magnets were measured via X‐ray photoelectron spectroscopy (XPS), as shown in **Figure** **3**. In addition, the XPS spectra of the Ce 3d level were processed by Advantage software (Thermo Fischer) to analyze the effect of Ce valence with increasing La substitution. The binding energy ranges corresponding to the sites of the Ce^3+^ and Ce^4+^ peaks were identified according to references.^[^ ^31^, ^32^, ^42^, ^43^ ^]^ The high‐resolution spectra of the Ce 3d photoelectron peak fitting results showed that the Ce^3+^ and Ce^4+^ ions coexisted in all samples (Figure 3a). To estimate the variations in the ratios of the Ce^3+^ ions with increasing La substitution, the proportion of trivalent and tetravalent peak areas in Ce 3d_5/2_ and Ce 3d_3/2_ was calculated, as shown in Figure 3b. With an increase in La substitution, the relative area ratio of the f^1^ peak (Ce^3+^) gradually increased, while the relative area ratio of the f^0^+f^2^ peak (Ce^4+^) gradually decreased. As a result, the ratio of Ce^3+^ ions increased from 48.5% in the *x* = 0 magnet to 53.7% in the *x* = 0.3 magnet. Combined with the XRD phase composition analysis results (Figure 2 and Table 1), the following two factors were responsible for the increase in the ratio of Ce^3+^ ions: the first factor was a decrease in the content of the REFe_2_ phase with the Ce^4+^ ions, and the second factor was that the expansion of the RE_2_Fe_14_B lattice caused by the La element promoted the transfer of the Ce‐valence to Ce^3+^, which was consistent with the results reported in previous studies.^[^ ^31^, ^32^ ^]^ Compared to the Ce^4+^ ions, the Ce^3+^ ions had one localized 4f moment. Therefore, the Ce‐valence to migrate toward Ce^3+^ in the RE_2_Fe_14_B phases of the magnet was beneficial for increasing the magnetic moment of the RE_2_Fe_14_B phase, which provided additional assistance for improving *B*_r_.

**Figure 4:**
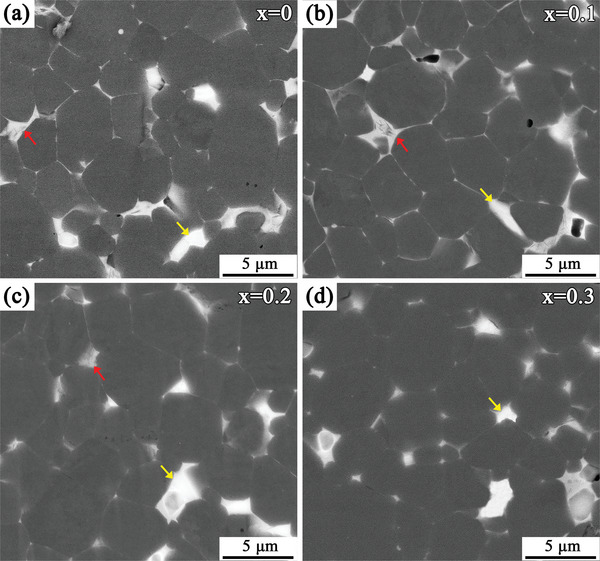
a–d) SEM images of the (La*_x_* Ce_1−_ *_x_*)‐40 (*x* = 0–0.3) magnets, where the red and yellow arrows represent two different intergranular phases.

The microstructure is an important factor that will restrict the magnetic properties of RE‐Fe‐B sintered magnets, and the distribution of lamellar GBs between 2:14:1 phase grains will seriously affect *H*_cj_.^[^ ^37^, ^44^ ^]^ The scanning electron microscope (SEM) images of the (La*_x_* Ce_1−_ *_x_*)‐40 (*x* = 0–0.3) magnets are shown in **Figure** **4**a–d, which were mainly composed of the RE_2_Fe_14_B phase grains with dark gray contrast and two RE‐rich intergranular phases with different contrasts (indicated by the red and yellow arrows). With increasing La substitution, the proportion of the light gray GB phase indicated by the red arrow gradually decreased. In the magnets with *x* ≥ 0.2 (Figure 4c,d), the light gray GB phases were significantly inhibited, and white GB phases dominated the intergranular regions. In addition, the distribution of a lamellar GB between the RE_2_Fe_14_B grains was also affected by an increase in La substitution. Compared to the *x* = 0 magnet (Figure 4a) with lamellar GBs between some of the RE_2_Fe_14_B grains, the continuity of lamellar GBs in the magnets with *x* = 0.1 (Figure 4b) improved. Evidently, this was conducive to improving *H*_cj_. A further increase in La substitution would deteriorate the continuity of lamellar GB. In the magnet with *x* = 0.2 (Figure 4c), the lamellar GBs were noticeably thinner and were only found in‐between a few RE_2_Fe_14_B grains. However, the RE‐rich phases were mainly distributed in large blocks in the *x* = 0.3 magnet (Figure 4d), and almost no continuous lamellar GBs were observed. Therefore, the gradual deterioration of lamellar GBs with *x* ≥ 0.2 La substitution was one of the reasons for the decrease in *H*_cj_.

**Figure 5:**
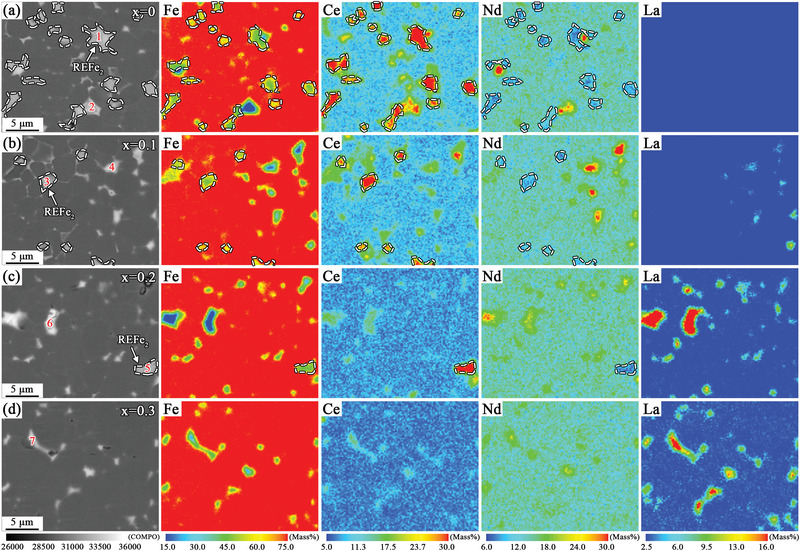
a–d) EPMA mapping images of the (La*_x_* Ce_1−_ *_x_*)‐40 (*x* = 0–0.3) magnets, where the intergranular phases marked by the dashed lines indicate the REFe_2_ phases.

To further clarify the effect of La substitution on element distribution, the Fe/Ce/Nd/La element mappings were obtained by performing electron probe microanalyzer (EPMA), as shown in **Figure** **5**. It was evident that the light gray GB phases (indicated by the white dashed area) mostly contained Ce and Fe elements, with a smaller amount of other RE elements. The wavelength dispersive X‐ray spectroscopy (WDS) results at the corresponding positions (Table S5, Supporting Information) showed that the light gray GB phases, as shown by the white dashed areas, had higher Fe content (>66 at%) and an atomic ratio of RE to Fe close to 1:2, which was the REFe_2_ phase. By contrast, the white GB phases had higher RE content and lower Fe content. The elemental distribution results intuitively confirmed that the content of the REFe_2_ phase in the triple junctions of the magnets gradually decreased with increasing La substitution, which was consistent with the results obtained by XRD refinement and SEM analysis. In addition, the increase in La substitution also led to the enrichment of La elements in the RE‐rich phase. When La substitution in the magnet was *x* = 0.2, a large number of La elements was enriched in the RE‐rich phase. By contrast, the content of La elements in the RE_2_Fe_14_B grains tested from the EPMA results was lower than the La proportion with nominal composition (Table S6, Supporting Information). The above results showed that the La element was more inclined to enter the RE‐rich phase, similar to the results reported in the literature.^[^ ^8^, ^9^, ^10^, ^16^ ^]^ Based on the SEM and EPMA results, we found that with *x* ≥ 0.2 La substitution, the deterioration of lamellar GBs was accompanied by a large enrichment of La elements in the RE‐rich phases. The rapid increase in the type II RE‐rich phase with a hexagonal structure (space group *P63/mmc*) in the XRD refinement results (Figure 2) was possibly directly related to the significant enrichment of La in the RE‐rich phase. To reveal the reason for the change in GB phase structures caused by the substitution of La elements, we next performed transmission electron microscopy (TEM) analysis of the triple junctions and lamellar GBs in the magnets.

**Figure 6:**
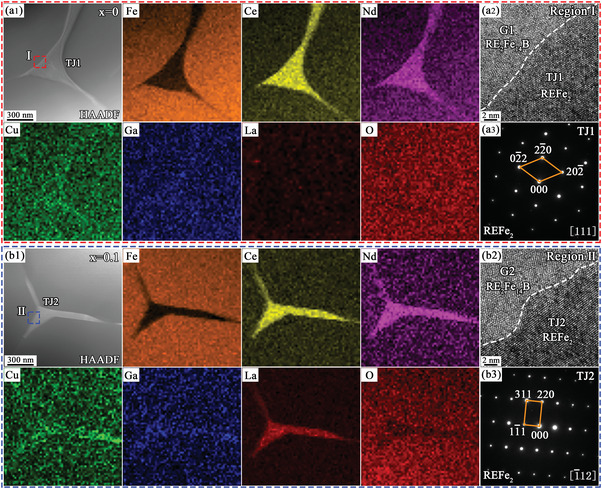
a1,b1) HAADF‐STEM images and element mapping of the REFe_2_ phases in the (La*_x_* Ce_1−_ *_x_*)‐40 (*x* = 0 and 0.1) magnets; a2,b2) HRTEM images of the red region I and blue region II in (a1) and (b1); a3,b3) SAED patterns of TJ1 and TJ2 in (a2) and (b2).

**Figure** **6** shows the TEM characterization and elemental mapping results of the REFe_2_ phases at the triple junctions in the *x* = 0 and 0.1 magnets. According to the high‐angle annular dark field‐scanning transmission electron microscopy (HAADF‐STEM) image (Figure 6a1) and the in situ element mapping results, we found that the Ce/Nd elements were enriched and the Fe element was poor in the TJ1 region (marked in Figure 6a1), while the other elements were consistent with the adjacent RE_2_Fe_14_B grains in the *x* = 0 magnet. The energy‐dispersive X‐ray spectroscopy (EDS) results (Table S7, Supporting Information) showed that the contents of Fe, Ce, and the other RE elements were 68.24, 23.67, and 6.98 at%, respectively, which was approximately the composition of the REFe_2_ phase. Figure 6a2 shows the high‐resolution TEM (HRTEM) image of the region I marked by red in Figure 6a1, indicating the interface between TJ1 and the RE_2_Fe_14_B grains. The selected area electron diffraction (SAED) of the TJ1 region (Figure 6a3) showed the [111] zone axis of the cubic REFe_2_ phase, supporting the EDS results. Figure 6b1 shows the HAADF‐STEM image of the triple junction (TJ2) in the *x* = 0.1 magnet, and the HRTEM image of the blue region II is shown in Figure 6b2, also indicating the interface between the TJ2 region and RE_2_Fe_14_B. The SAED pattern of the TJ2 region (Figure 6b3) revealed the [−112] zone axis of the cubic REFe_2_ phase, which was also confirmed by the EDS results (Table S7, Supporting Information). Interestingly, the element mapping and EDS results showed that the REFe_2_ phase in the TJ2 region had a small enrichment of La and Cu/Ga elements. The EDS results (Table S7, Supporting Information) showed that the REFe_2_ phase in the TJ2 region had lower Fe and Ce content than the TJ1 region, and the content of La elements also reached 0.98 at%. Meanwhile, the content of Cu and Ga elements in the TJ2 region was higher than that in the TJ1 region, increasing from 0.55 and 0.56 at% to 1.64 and 1.37 at%, respectively. Previous studies have shown that the REFe_2_ phase had lower Cu content compared to the conventional RE‐rich phase in the Nd‐Ce‐Fe‐B magnets.^[^ ^17^, ^19^, ^37^ ^]^ However, by doping (Nd, Pr)H*_x_* and Cu, the Cu‐dissolved REFe_2_ phase with higher Nd/Pr and lower Ce content appeared in the intergranular regions, which was conducive to the formation of lamellar GBs with better continuity.^[^ ^37^ ^]^ The above findings demonstrated that a comparable effect was generated in the La‐substituted magnets, which was beneficial to *H*_cj_ by optimizing the distribution of lamellar GBs.

**Figure 7:**
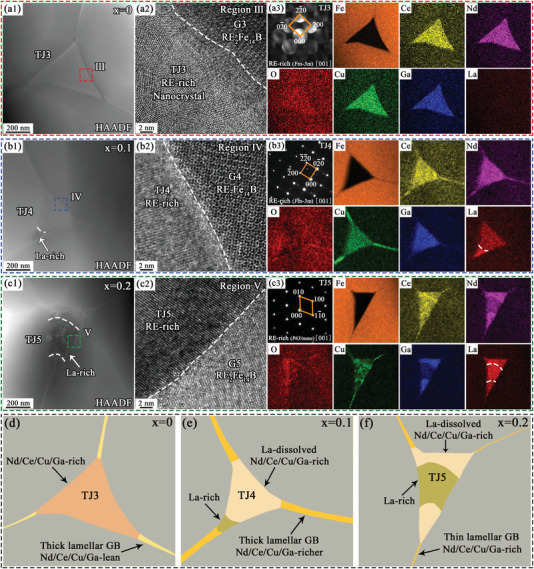
a1–c1) HAADF‐STEM images and element mapping of the RE‐rich phases in the (La*_x_* Ce_1−_ *_x_*)‐40 (*x* = 0, 0.1, and 0.2) magnets; a2–c2) HRTEM images of the corresponding regions III–V in (a1)–(c1); a3–c3) NBD patterns of TJ3 and SAED patterns of TJ4 and TJ5; d–f) schematic diagram of the triple junctions and adjacent lamellar GBs in the three magnets.

The RE‐rich phases at the triple junctions in the (La*_x_* Ce_1−_ *_x_*)‐40 (*x* = 0, 0.1, and 0.2) magnets were also characterized by TEM. The HAADF‐STEM image (**Figure** **7**a1) of the RE‐rich triple junction (TJ3) and the HRTEM image in region III marked in red (Figure 7a2) for the *x* = 0 magnet revealed a fuzzy interface between the TJ3 and RE_2_Fe_14_B grains, where the TJ3 region was composed of nanocrystals. By calibrating the nanobeam electron diffraction (NBD) patterns (Figure 7a3) of TJ3, the fcc‐structured RE‐rich phase with a [001] zone axis was present. The elemental mapping results showed that the TJ3 region had a higher Ce/Nd concentration and a lower Fe concentration than the adjacent RE_2_Fe_14_B grains. More evidently, Cu and Ga elements were significantly enriched in the TJ3 region, which differed from the REFe_2_ phase (TJ1 in Figure 6a1). Figure 7b1 shows the HAADF‐STEM image of the RE‐rich triple junction (TJ4) in the *x* = 0.1 magnet, while Figure 7b2 shows the HRTEM image of blue region IV. Unlike TJ3, TJ4 was composed of a single crystal and had a clear interface with RE_2_Fe_14_B grains. Furthermore, it also belonged to the RE‐rich phase of the fcc‐structure with the [001] zone axis, according to the SAED pattern (Figure 7b3). In addition to the enrichment of the Ce/Nd and Cu/Ga elements, the elemental mapping results demonstrated that the concentration of the La element was also significantly higher than that of adjacent RE_2_Fe_14_B grains. Interestingly, unlike the uniform distribution of Ce/Nd/Cu/Ga elements in TJ3, the distribution of RE/Cu/Ga elements in TJ4 was segregated due to the addition of the La element. At the corner of the triple junction (TJ4), a Ce/Nd/Cu/Ga‐lean and La‐rich region appeared, as indicated by the white arrow in Figure 7b1. More importantly, this promoted more Ce/Nd/Cu/Ga elements to flow into the lamellar GB between the adjacent RE_2_Fe_14_B grains. When La substitution in the magnet increased to 0.2, the proportion of La‐rich region in the RE‐rich triple junction (TJ5) was higher, as shown by the white arrow in Figure 7c1. In addition, the elemental mapping results demonstrated a substantial decrease in the concentration of Cu/Ga elements in this La‐rich region. The HRTEM image (Figure 7c2) of the green region V in Figure 7c1 showed that the La‐rich region in TJ5 consisted of a single crystal, and the SAED pattern (Figure 7c3) indicated that it belonged to a hexagonal structure with a [001] zone axis. The effect of La substitution on the RE‐rich phase in both the triple junctions and lamellar GBs is more intuitively shown by the schematics in Figure 7d,f. In the magnet with appropriate La substitution (*x* = 0.1) (Figure 7e), fcc‐structured RE‐rich TJ4 with a higher RE/Cu/Ga concentration and a lower Fe concentration had better wettability with the RE_2_Fe_14_B grains. However, the La element led to a segregation effect of the RE/Cu/Ga elements. Therefore, a clearer and continuous thick lamellar GB rich in Ce/Nd/Cu/Ga extended from TJ4 to the interface between the adjacent RE_2_Fe_14_B grains. By contrast, the lamellar GB extending from the Ce/Nd/Cu/Ga‐rich TJ3 to the adjacent RE_2_Fe_14_B grains in the *x* = 0 magnet had a higher Fe concentration and a lower Ce/Nd/Cu/Ga concentration, and the thickness of the lamellar GB also decreased (Figure 7d). However, in the magnet with excessive La substitution (*x* = 0.2) (Figure 7f), a large blocky La‐rich region with a hexagonal structure appeared in the RE‐rich triple junction (TJ5), which led to a thinner thickness of the lamellar GB and lower concentrations of Ce/Nd/Cu/Ga elements. Evidently, the appearance of large blocky La‐rich regions with a hexagonal structure in the RE‐rich triple junctions was also the direct reason for the rapid increase in the mass fraction of the type II RE‐rich phase with a hexagonal structure (space group *P63/mmc*) in the *x* ≥ 0.2 magnets (Figure 2).

**Figure 8:**
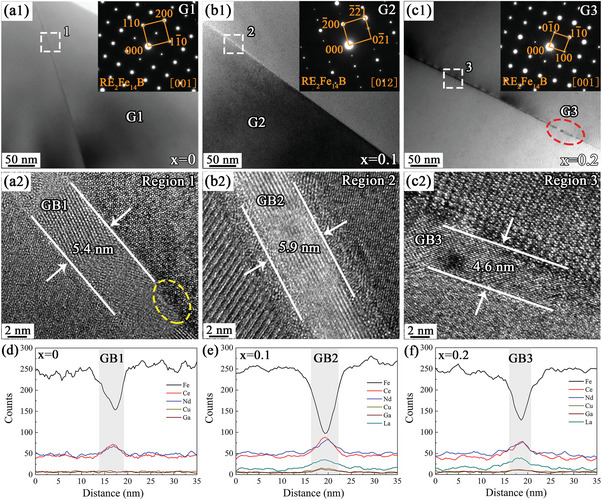
TEM characterization of thin lamellar GB of the (La*_x_* Ce_1−_ *_x_*)‐40 (*x* = 0, 0.1, and 0.2) magnets; a1–c1) BFI of the three types of magnets; a2–c2) HRTEM images of corresponding GB positions 1–3; d–f) line scanning results of GB1–GB3.

TEM characterization in **Figure** **8** further revealed the evolution of lamellar GBs in the magnets with La substitution. Figure 8a1–c1 shows the bright field images (BFIs) of the three magnets obtained along the [001], [012], and [001] zone axes of G1–G3, respectively, showing lamellar GBs between the adjacent RE_2_Fe_14_B grains. The HRTEM images of the corresponding white dashed regions 1–3 are shown in Figure 8a2–c2. Compared to the thickness of the lamellar GB (≈5.4 nm in size) in the *x* = 0 magnet, the thickness of the continuous lamellar GB in the *x* = 0.1 magnet increased slightly to 5.9 nm. In addition, the lamellar GB transitioned from nanocrystals to single crystals, which was also possibly related to the different crystal structures in the RE‐rich triple junctions of the *x* = 0 and 0.1 magnets (Figure 7a,b). However, with a further increase in La substitution, the continuity of the lamellar GB was destroyed, as shown in the red dashed region in Figure 8a3. In addition, the thickness of the continuous lamellar GB between some regions also decreased progressively. The thickness of the lamellar GB in the *x* = 0.2 magnet decreased to 4.6 nm, as shown in Figure 8b3, and in the *x* = 0.3 magnet, the thickness of the lamellar GB decreased to 2.6 nm (Figure S5, Supporting Information). Furthermore, line scan analysis of the lamellar GB in the *x* = 0, 0.1, and 0.2 magnets was performed, and the results are shown in Figure 8d,f. Compared to the Fe‐rich lamellar GB in the *x* = 0 magnet (Figure 8d), the lamellar GB in the *x* = 0.1 magnet had a lower Fe concentration and a higher Cu/Ga concentration (Figure 8e). With excessive La substitution in the *x* = 0.2 magnet (Figure 8f), although Cu/Ga elements were also enriched in the lamellar GB, the concentration of Fe elements was higher than that in the *x* = 0.1 magnet. The aforementioned variations in the lamellar GBs were mostly caused by variations in the compositions and structures of the triple junctions in the magnets. In the x = 0 magnet, most of the triple junctions consisted of the REFe_2_ phase with a higher Fe concentration and a lower RE/Cu/Ga concentration, as shown in Figure 6a. In addition, the lamellar GB extending from the RE/Cu/Ga‐rich triple junction (TJ3) to the adjacent RE_2_Fe_14_B grains had a higher Fe concentration (Figure 7a). As a result, the above two reasons led to a lamellar GB with a higher Fe concentration, as shown in Figure 8a. In the *x* = 0.1 magnet, there were two significant changes in the triple junctions by La substitution, which was the reason for the formation of a thicker and continuous lamellar GB with a lower Fe concentration, as shown in Figure 8b; the first involved inhibition of the REFe_2_ phase and optimization of its composition (Figure 6b), while the second was that the La element in the RE‐rich triple junction promoted the segregation of RE/Cu/Ga elements, resulting in more Ce/Nd/Cu/Ga elements flowing into the lamellar GB (Figure 7b). With excessive La substitution, although the REFe_2_ phase was suppressed, large blocky La‐rich regions with a hexagonal structure appeared in the RE‐rich triple junctions, resulting in a decrease in the concentration of Ce/Nd/Cu/Ga elements in the adjacent thinner lamellar GB (Figure 7c). This was also the reason for the decrease in thickness and increase in Fe concentration in the lamellar GB in Figure 8c. In the 2:14:1‐type sintered magnets, the defect areas at the grain boundaries and grain surfaces were the preferential nucleation sites of the reversed domains.^[^ ^45^, ^46^ ^]^ In addition, the magnetic isolation effect of non‐ferromagnetic or weakly ferromagnetic thin lamellar GB between the 2:14:1 phase grains was an important factor affecting *H*_cj_.^[^ ^47^ ^]^ As shown by the yellow dashed region in Figure 8a2, the *x* = 0 magnet had a defect region at the interface between the lamellar GB and the main phase grains. In addition, there was a higher Fe concentration in the lamellar GB. As a result, once the reversed domains nucleated in the local defect region, they would rapidly expand to adjacent grains, which was harmful to *H*_cj_. However, the lamellar GB of the *x* = 0.1 magnet had a lower Fe concentration and an additional increase in thickness, which could better suppress ferromagnetic coupling between the adjacent grains and enhance *H*_cj_. By contrast, when the amount of La substitution further increased, the thickness of the thin lamellar GB gradually decreased, and the Fe element concentration started to increase. This meant that the ability to suppress ferromagnetic coupling was significantly reduced, which inevitably led to a decrease in *H*_cj_.

### 2.3 Characterization of Magnetization Reversal Behavior

**Figure 9:**
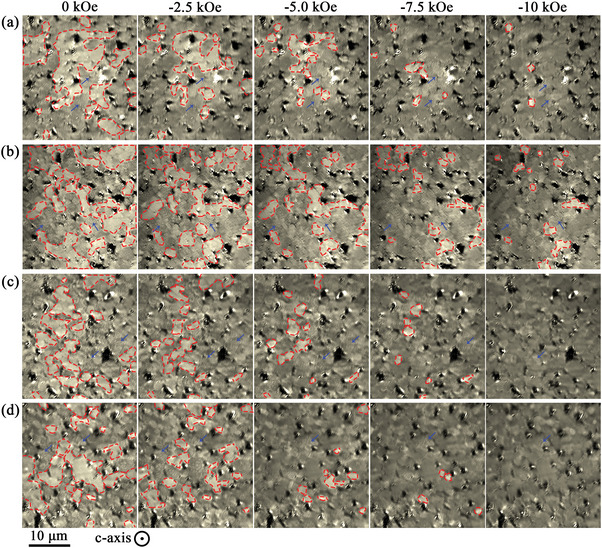
a–d) Domain evolution of the (La*_x_* Ce_1−_ *_x_*)‐40 (*x* = 0–0.3) magnets during the magnetization reversal process, in which the positive single‐domain regions are indicated by red dashed areas, and the maze‐like multidomains are indicated by the blue arrows. (The observing plane is perpendicular to *c*‐axis.)

**Figure** **9**a–d shows the domain evolution of the (La*_x_* Ce_1−_ *_x_*)‐40 (*x* = 0–0.3) magnets during the magnetization reversal process. Before observations, the magnets were magnetized to saturation along the *c*‐axis in a pulsed magnetic field of 100 kOe. To observe the magnetization reversal process of the four magnets, the magnetic field perpendicular to the observation plane gradually increased from 0 to −10 kOe. Due to the demagnetization effect of the samples, numerous maze‐like multidomains (shown as blue arrows) formed in the remanence state (0 kOe), and some positive single‐domain regions with brighter contrast (indicated by red dashed areas) were also observed. In addition, the black contrast regions in the figure were the intergranular phases. Compared to the *x* = 0 magnet, the *x* = 0.1 magnet had a larger proportion of positive single‐domain regions in the remanence state. However, the proportion of positive single‐domain regions in the remanence state of the magnets gradually decreased as La substitution further increased. With an increase in the reverse magnetic field, the proportion of positive single‐domain regions with a brighter contrast gradually decreased, while the proportion of maze‐like multidomain regions with darker contrast quickly increased. By contrast, the *x* = 0.1 magnets exhibited the highest proportion of positive single domains for the same applied magnetic field. Meanwhile, the distribution of positive single‐domain regions was more dispersed, indicating that the thin lamellar GB in the magnet had a better magnetic isolation effect. Under an applied magnetic field of −10 kOe, the magnets with *x* = 0.1 also had more positive single‐domain regions than the *x* = 0 magnets. Meanwhile, most of the grains in the *x* = 0.2 and 0.3 magnets reached the reversed saturation, while the remaining small portion of the grains was still maintained in a maze‐like multidomain state (indicated by the blue arrow). The above domain evolution results revealed that the *x* = 0.1 magnets had the strongest resistance to the applied magnetic field, which guaranteed a higher *H*_cj_ than the other magnets.

**Figure 10:**
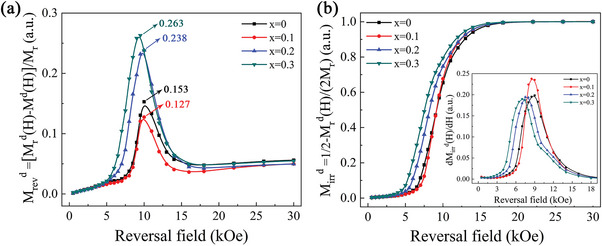
a,b) Reversible and irreversible portions during the magnetization reversal process of the (La*_x_* Ce_1−_ *_x_*)‐40 (*x* = 0–0.3) magnets (the inset shows the first derivative curves of the irreversible portions).

To further analyze the magnetization reversal behavior of the (La*_x_* Ce_1−_ *_x_*)‐40 (*x* = 0–0.3) magnets, we tested the recoil loops of the four magnets (Figure S6, Supporting Information). During the magnetization reversal process, the reversible [*m*_rev_^d^(H) = (*M*_r_^d^(H) − *M* ^d^(H))/*M*_r_] and irreversible [*m*_irr_^d^(H) = 1/2 − *M*_r_^d^(H)/(2*M*_r_)] portion dependence of the reversal field (*H*) was obtained by the recoil loops, as shown in **Figure** **10**a,b. The reversible and irreversible portions of the magnetization reversal process reflected the ferromagnetic coupling strength between the grains and the distribution of effective anisotropy.^[^ ^48^, ^49^, ^50^ ^]^ The maximum reversible portion of the *x* = 0.1 magnet decreased to 12.7% of the remanence compared to the *x* = 0 magnet, with a maximum reversible portion of 15.3% of the remanence (Figure 10a). However, when the amount of La substitution was further increased to 0.2 and 0.3, the maximum reversible portion increased sharply to 23.8% and 26.3% of the remanence, respectively. This indicated that in the *x* = 0.1 magnet, the ferromagnetic coupling strength was significantly weakened due to the formation of continuous lamellar GB with a low Fe concentration between most of the RE_2_Fe_14_B grains (Figures 4b and 8b). However, the deterioration of the lamellar GB with increasing La substitution (*x* ≥ 0.2) resulted in a higher maximum reversible portion in the magnets. Figure 10b shows the reversal field dependence of the irreversible portions. As the reversal field increased, the irreversible portion of the four magnets slowly increased. The irreversible portions started to quickly increase as soon as the reversal field reached the irreversible nucleation field (*H*_n_), because some of the grains started to reverse. Interestingly, the *H*_n_ of the magnets increased first and then declined with increasing La substitution, resulting in the largest *H*_n_ acquired in the *x* = 0.1 magnet. Meanwhile, the first derivative curves of the irreversible portions (inset in Figure 10b) also clearly showed that an obvious increase in the irreversible portion of the *x* = 0.1 magnet was the most difficult. This indicated that the reversed domains in the *x* = 0.1 magnet had more difficulty nucleating and expanding. Therefore, the *x* = 0.1 magnet had a larger proportion of positive single‐domain regions in the remanence state and exhibited the strongest resistance to the reversal field during the magnetization reversal process (Figure 9b). Furthermore, the peak‐type first derivative curves also showed the narrowest peak width at the half height for the *x* = 0.1 magnet, supporting the best squareness of the demagnetization curve for the *x* = 0.1 magnet.

## 3 Discussion

A series of interesting alterations affecting the magnetic properties occurred in the magnets after La replaced Ce. The mass fraction of the RE_2_Fe_14_B phase and the RE‐rich phase gradually increased with the substitution of La for Ce, while the mass fraction of the REFe_2_ phase gradually decreased. Because the La elements tended to enter the RE‐rich phase, this led to actual La substitutions, *x*, of 0.066, 0.183, and 0.285 (Table S6, Supporting Information) in the RE_2_Fe_14_B phase for the magnets with 0.1, 0.2, and 0.3 nominal La substitutions, respectively. The entry of La atoms into the RE_2_Fe_14_B phase was beneficial for increasing *M*_s_, followed by an increase in *B*_r_. In addition, the expansion of the RE_2_Fe_14_B phase lattice caused by the large radius La atoms increased the Ce^3+^ ion ratio in the RE_2_Fe_14_B phases (Figure 3), which improved the *B*_r_ further. When La entered the RE_2_Fe_14_B phase, it also weakened *H*_A_, which caused a decrease in *H*_cj_. Surprisingly, the *H*_cj_ with appropriate La substitution (*x* = 0.1) increased considerably. The *H*_cj_ of the magnet was not only restricted by the *H*_A_ of the RE_2_Fe_14_B phase but was also affected by the composition and distribution of the RE‐rich phases. Appropriate La substitution also optimized the composition and structure of the RE‐rich phase, which was the key factor for increasing *H*_cj_, breaking through the remanence–coercivity trade‐off and achieving simultaneous improvement of *B*_r_ and *H*_cj_.

**Figure 11:**
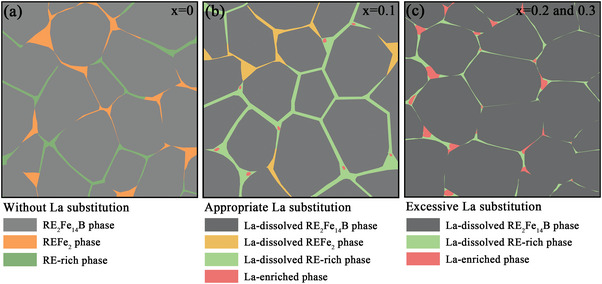
Microstructure diagrams of the (La*_x_* Ce_1−_ *_x_*)‐40 (*x* = 0–0.3) magnets a) without La substitution (*x* = 0), b) appropriate La substitution (*x* = 0.1), and c) excessive La substitution (*x* = 0.2 and 0.3).

Based on the above microstructure characterizations, we constructed microstructure diagrams of the magnets with different La substitutions, as shown in **Figure** **11**a–c. In the magnets with an appropriate amount of La substitution (Figure 11b), the proportion of the REFe_2_ phase in the intergranular region dramatically decreased, while a decrease in the concentration of Fe element and a small enrichment of La/Cu/Ga elements in the REFe_2_ phase occurred (Figure 6b). These composition changes in the REFe_2_ phase improved wettability and assisted in the formation of lamellar GBs at the interface between adjacent grains.^[^ ^37^ ^]^ Moreover, appropriate La substitution was conducive to forming the fcc‐structure RE‐rich triple junction with higher RE/Cu/Ga element content in the magnets. This type of triple junction had good wettability,^[^ ^38^, ^39^, ^40^ ^]^ which encouraged the formation of large‐area, continuous, and thick lamellar GBs (Figures 4, 7, and 8b). More importantly, the enriched La in the RE‐rich triple junctions led to sizable segregation in the distribution of the RE/Cu/Ga elements, prompting more Ce/Nd/Cu/Ga elements to migrate into the lamellar GBs (Figures 7b and 8b). In comparison, in the magnet without La substitution (Figure 11a), the REFe_2_ phase dominated the intergranular regions (Figure 5a). Due to high Fe and lower RE/Cu/Ga concentrations in the REFe_2_ phase (Figure 6a), poor wettability led to its mostly agglomerative distribution, with poor continuity and a high Fe concentration even in the partially thin lamellar GBs that formed. In addition, the enrichment of the RE/Cu/Ga elements and the reduction of Fe element concentration were also observed in the RE‐rich triple junctions without La substitution (Figure 7a). Due to the absence of La elements promoting the segregation effect, the lamellar GBs extending from the RE‐rich triple junctions still had a lower concentration of Ce/Nd/Cu/Ga elements and a higher concentration of Fe elements (Figures 7a and 8a). The absence of Ce/Nd/Cu/Ga elements led to the deterioration of wettability, which was detrimental to the formation of large‐area, continuous lamellar GBs (Figure 4a). For the magnet with excessive La substitution (*x* ≥ 0.2) (Figure 11c), the intergranular phases in the magnets were mainly composed of RE‐rich phases, while the REFe_2_ phase disappeared. However, the high content of La substitution led to large‐block hexagonal structure La‐rich phases with a low concentration of Ce/Nd/Cu/Ga elements in the RE‐rich triple junctions (Figure 7c), resulting in the deterioration of wettability. Therefore, the continuity of the lamellar GB extending from the triple junction was also poor (Figures 4 and 8). This was similar to previous studies, which found that a large number of hexagonal La‐rich oxide phases were generated at the triple junctions after La replaced Nd, destroying the wettability of the GB phases and leading to the deterioration of the continuous lamellar GBs.^[^ ^9^, ^51^ ^]^ Due to the low oxygen content of the magnet (<650 ppm), the content of RE oxides in the intergranular phase of the magnets in our study was low, as reflected also by the O element mapping results (Figures 6 and 7), and the hexagonal La‐rich phase could be retained.

The substitution of Ce with an appropriate amount of La in the sintered RE‐Fe‐B magnet resulted in two significant changes in the intergranular phases, which consisted of two scenarios for achieving higher *H*_cj_; the first involved decreasing the REFe_2_ phase and optimizing its composition to obtain better wettability, while the second involved promoting the formation of large‐area, continuous lamellar GBs with lower Fe content and higher Ce/Nd/Cu/Ga concentration. As a result, the lamellar GBs of the magnet had a stronger magnetic isolation effect, which led to the magnet under the same reversal field exhibiting more positive domains, and the distribution was more uniform (Figure 9b). Therefore, the *H*_cj_ of the sintered magnets was enhanced, even though the *H*_A_ of the RE_2_Fe_14_B grains decreased. By contrast, the magnet without La substitution had a high content of the REFe_2_ phase, and the higher Fe concentration in the lamellar GBs led to the deterioration of the magnetic isolation effect, which was not conducive to suppressing the propagation of the reversed domains. Meanwhile, the defects at the lamellar GBs in the magnet without La substitution could cause the reversed domains to nucleate more easily, resulting in low *H*_cj_. Therefore, the magnet had a large proportion of reversed domains in the remanence state, and the reversed domains expanded rapidly with an increase in the reversal field (Figure 9a). Evidently, even if the RE_2_Fe_14_B grains had a higher *H*_A_, this would lead to lower *H*_cj_ of the sintered magnets. With an excessive substitution amount of La, the *H*_A_ of the RE_2_Fe_14_B grains decreased significantly. Although the REFe_2_ phase was suppressed, the appearance of large‐block hexagonal structure La‐rich phases in the triple junctions deteriorated the continuity of the lamellar GBs. Under a lower reversal field, the reversed domains could more easily nucleate and propagate (Figure 9c,d), resulting in a sharp decrease in the *H*_cj_ of the sintered magnets. This finding was supported by the reported facts that ^[^ ^52^, ^53^, ^54^, ^55^ ^]^ the *H*_cj_ values of the LaCe‐containing magnets prepared using the La_35_Ce_65_ alloy were lower than that of the Ce‐containing magnets. In addition, the La atoms that entered the RE_2_Fe_14_B phase were beneficial for improving the *B*_r_ and temperature stability of the magnets, and the resulting lattice expansion promoted an increase in the Ce^3+^ ion ratio, which provided additional benefit for improving *B*_r_. Consequently, the *H*_cj_, *B*_r_, (*BH*)_max_, and temperature stability were simultaneously enhanced in the x = 0.1 magnet. Notably, we successfully achieved the same findings with the (La*_x_* Ce_1−_ *_x_*)‐60 magnets (Figure S7, Supporting Information), and demonstrating our strategy, the substitution of an appropriate amount of La for Ce was not limited to specific components of the (La*_x_* Ce_1−_ *_x_*)‐40 magnets, supporting the successful solution of the remanence‐coercivity trade‐off in sintered RE‐Fe‐B magnets with high‐abundance RE, i.e., the simultaneous enhancement of *B*_r_ and *H*_cj_ of sintered magnets with high‐abundance RE. The above findings not only revealed a correlation between the microstructure and properties in La‐substituted Nd‐Ce‐Fe‐B magnets but also clarified the influence mechanism of the REFe_2_ and RE‐rich phases as intergranular phases on the magnetic properties. As a novel discovery to overcome trade‐off between remanence and coercivity, the replacement of Ce with an appropriate amount of La may offer new opportunities for the comprehensive utilization of La‐Ce elements and the development of high‐performance RE‐Fe‐B sintered magnets.

## 4 Conclusions

In summary, we realized the simultaneous improvement of *H*_cj_, *B*_r_, and (*BH*)_max_, and temperature stability by substituting La for Ce to synergistically regulate the phase composition, Ce‐valence, and GB phase structure in magnets. The main conclusions were as follows. 1) Partial La atoms that entered the RE_2_Fe_14_B phase were beneficial for improving the *B*_r_ and temperature stability of the magnets, and the resulting lattice expansion promoted the Ce^3+^ ion ratio, which provided additional benefit for *B*_r_. 2) Due to the high Fe and low RE/Cu/Ga concentrations in the REFe_2_ phase, the poor wettability led to its mostly agglomerative distribution, with poor continuity and a higher Fe concentration even in the partially lamellar GBs that formed, which was detrimental to *H*_cj_. 3) In the magnets (*x* = 0.1) with an appropriate amount of La substitution, the La/Cu/Ga‐dissolved REFe_2_ phase with higher Nd and lower Ce/Fe appeared in the triple junctions, and its mass fraction also significantly decreased. More significantly, the enriched La in the RE‐rich triple junctions led to sizable segregation in the distribution of RE/Cu/Ga elements, prompting the formation of Ce/Nd/Cu/Ga‐rich continuous thicker lamellar GBs, which had a stronger magnetic isolation effect. As a result, the *H*_cj_ was enhanced, even though the *H*_A_ of the RE_2_Fe_14_B grains decreased. 4) For the magnets with excessive La substitution (*x* ≥ 0.2), although the REFe_2_ phase was suppressed, the appearance of large‐block La‐rich phases with a hexagonal structure in the triple junctions deteriorated the continuity of the thinner lamellar GBs. Meanwhile, the *H*_A_ of the RE_2_Fe_14_B grains decreased significantly, resulting in a sharp decrease in *H*_cj_. This work provides a new strategy for overcoming the trade‐off between remanence and coercivity in LaCe‐based sintered magnets based on clarifying the influence mechanism of La elements.

## 5 Experimental Section

5.1

5.1.1

5.1.1.1

Alloys with a nominal composition of [(La*_x_* Ce_1−_ *_x_*)_0.4_Nd_0.4_Pr_0.2_]_31_Fe_bal_M_1.95_B_0.96_ (*x* = 0, 0.1, 0.2, and 0.3, M = Al, Cu, Ga, Co, and Zr in wt%) (hereafter denoted as (La*_x_* Ce_1−_ *_x_*)‐40) were prepared by a strip‐casting (SC) method. Four fine powders with an average particle size X_50_ of about 2.9 µm were prepared by hydrogen decrepitation (HD) and the jet milling (JM) method. In addition, lubricants and antioxidants were added before and after JM to improve the magnetic properties. Alignment and compaction were performed in a 1.8 T magnetic field, followed by isostatic pressing at 225 MPa of pressure to obtain the compacts. Under the protection of Ar_2_ gas, the compacts were maintained at 1030–1070 °C for 3 h to explore the optimal sintering temperature. Subsequently, to produce the magnets with the best performance, two‐stage annealing was carried out at 805–865 °C for 3 h and at 380–450 °C for 4 h.

The demagnetization curves of the magnets with different La substitutions at different temperatures were obtained using a permanent magnet measuring system (NIM‐500C), and a vibrating sample magnetometer (VSM, VersaLab, Quantum Design) was used to measure the recoil loops of the four magnets at 20 °C. The phase constituents and alignment degrees of the magnets with different La substitutions were measured by XRD with Cu Ka radiation (Ultima IV, Rigaku), and Rietveld refinements were carried out. The microstructures of the magnets with different La substitutions were observed by using a SEM (NOVA‐NANO‐200, FEI). The triple junctions and lamellar GBs of the magnets with different La substitutions were observed by using a TEM (Tecnai F30, FEI) equipped with EDS. The elemental distribution maps of magnets with different La substitutions were assessed by using an EPMA (JXA‐800, JEOL) with WDS, and the chemical states of Ce in four types of magnets were measured via XPS (Escalab 250Xi). A magneto‐optical Kerr microscope (MOKE, BH‐786IP‐PK, NEOARK) was used to investigate the process of magnetization reversal in the magnets with different La substitutions.

## Conflict of Interest

The authors declare no conflict of interest.

## Supporting information

Supporting Information

## Data Availability

The data that support the findings of this study are available from the corresponding author upon reasonable request.
