# Supplementary material for: Strategy for Co‐Enhancement of Remanence–Coercivity of RE‐Fe‐B Sintered Magnets with High Ce‐Content: Appropriate La Substitution
Source: Adv Sci (Weinh). 2023 Apr 17;10(16):2301312. doi: 10.1002/advs.202301312 (PMC10238191; doi:10.1002/advs.202301312)
Supplement: Supplementary file 1 — Supporting Information [file ADVS-10-2301312-s001.pdf]

Strategy for co-enhancement of remanence-coercivity of RE-Fe-B sintered magnets with high Ce-content: appropriate La substitution

Hao Chen, Yuqing Li, Hongguo Zhang, Weiqiang Liu, Haihui Wu, Yuan Qin, Ming Yue*, Qifeng Wei, Baoguo Zhang, Jinghui Di*

H. Chen[1], Y. Li[1], H. Zhang, W. Liu, H. Wu, Y. Qin, M. Yue
Faculty of Materials and Manufacturing, Key Laboratory of Advanced Functional Materials, Ministry of Education of China
Beijing University of Technology
Beijing 100124, China
E-mail: liuwq@bjut.edu.cn; yueming@bjut.edu.cn

Q. Wei, B. Zhang
Hangzhou Foresee Technology Co., Ltd
Hangzhou 311500, China

J. Di
Hangzhou Magmax Technology Co., Ltd
Hangzhou 311500, China

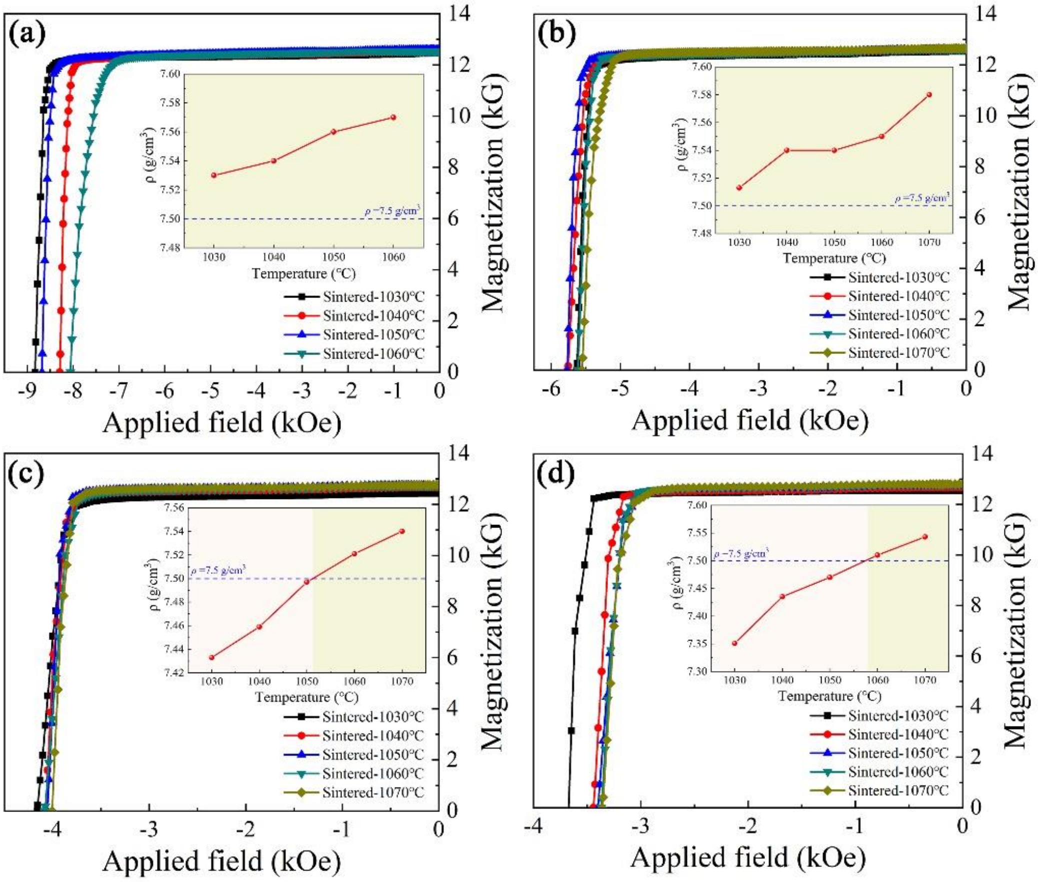

Figure S1. Room-temperature demagnetization curves of (La_xCe_{1-x})-40 (x = 0–0.3) magnets at different sintering temperatures, (a)-(d) corresponding to x = 0–0.3, respectively, (the illustration shows the sintering temperature dependence of density).

Table S1. The magnetic properties of the (La_xCe_{1-x})-40 (x = 0–0.3) magnets at different sintering temperatures.

La content (x)	temperature (°C)	B_r (kG)	H_{cj} (kOe)	$(BH)_{max}$ (MGOe)	H_k/H_{cj} (%)	ρ (g/cm^3)
0	1030°C	12.46	8.83	37.84	97.0	7.53
	1040°C	12.54	8.29	38.01	97.1	7.54
	1050°C	12.60	8.68	38.30	97.2	7.56
	1060°C	12.53	8.06	38.22	91.5	7.57
0.1	1030°C	12.52	5.63	36.01	96.6	7.51
	1040°C	12.60	5.76	36.57	94.8	7.54
	1050°C	12.63	5.77	37.19	96.6	7.54
	1060°C	12.63	5.61	36.89	96.1	7.55
	1070°C	12.65	5.56	36.67	93.5	7.58
0.2	1030°C	12.43	4.16	30.67	92.2	7.43
	1040°C	12.66	4.07	31.95	94.3	7.46
	1050°C	12.73	4.08	31.55	92.6	7.50
	1060°C	12.76	4.06	32.30	94.7	7.52
	1070°C	12.76	4.01	32.08	95.0	7.54
0.3	1030°C	12.53	3.67	28.16	94.4	7.35
	1040°C	12.66	3.44	28.88	93.4	7.44
	1050°C	12.73	3.40	28.23	92.6	7.47
	1060°C	12.79	3.37	28.64	93.6	7.51
	1070°C	12.80	3.36	27.82	92.2	7.54

According to the magnetic properties and density test results in Table S1, the best sintering temperatures of (La_xCe_{1-x})-40 (x=0, 0.1, 0.2, and 0.3) magnets are 1050°C, 1050°C, 1060°C, and 1060°C, respectively.

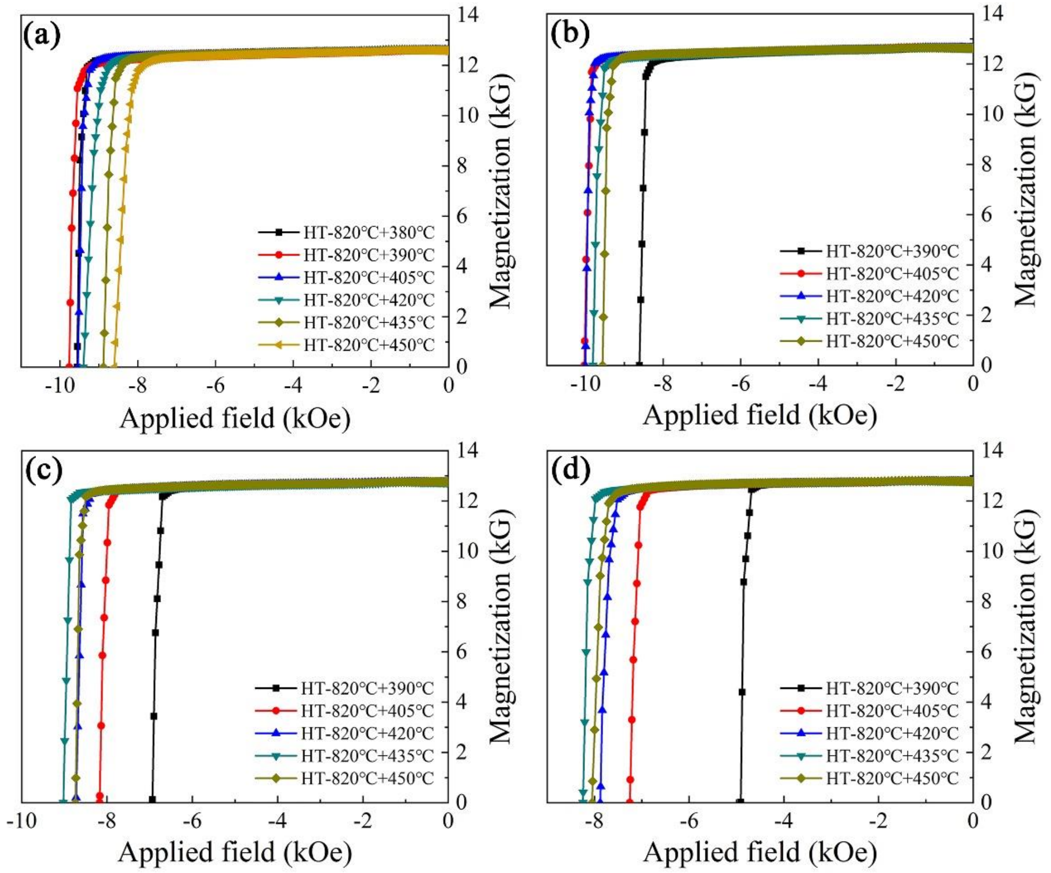

Figure S2. Room-temperature demagnetization curves of (La_xCe_{1-x})-40 (x = 0–0.3) magnets at different annealing temperatures, (a)-(d) corresponding to x = 0–0.3, respectively.

Table S2. The magnetic properties of the (La_xCe_{1-x})-40 (x = 0–0.3) magnets at different annealing temperatures.

La content (x)	Temperatures (°C)	B_r (kG)	H_{cj} (kOe)	$(BH)_{max}$ (MGOe)	H_k/H_{cj} (%)
0	820°C+380°C	12.59	9.56	38.59	97.7
	820°C+390°C	12.58	9.76	38.13	97.2
	820°C+405°C	12.60	9.54	38.70	97.2
	820°C+420°C	12.62	9.40	38.89	94.6
	820°C+435°C	12.60	8.89	38.72	96.5
	820°C+450°C	12.58	8.60	38.32	94.0
0.1	820°C+390°C	12.63	8.61	38.36	98.1
	820°C+405°C	12.64	10.03	38.93	98.2
	820°C+420°C	12.64	10.01	38.77	98.0
	820°C+435°C	12.63	9.81	38.75	97.2
	820°C+450°C	12.63	9.56	38.92	97.5
0.2	820°C+390°C	12.74	6.94	39.27	96.9
	820°C+405°C	12.73	8.17	39.56	97.4
	820°C+420°C	12.74	8.73	39.58	98.1
	820°C+435°C	12.74	9.02	39.34	98.1
	820°C+450°C	12.75	8.74	39.60	97.7
0.3	820°C+390°C	12.77	4.91	36.58	96.2
	820°C+405°C	12.78	7.25	39.47	97.0
	820°C+420°C	12.77	7.88	39.63	95.9
	820°C+435°C	12.78	8.24	39.82	97.2
	820°C+450°C	12.77	8.05	39.84	95.9

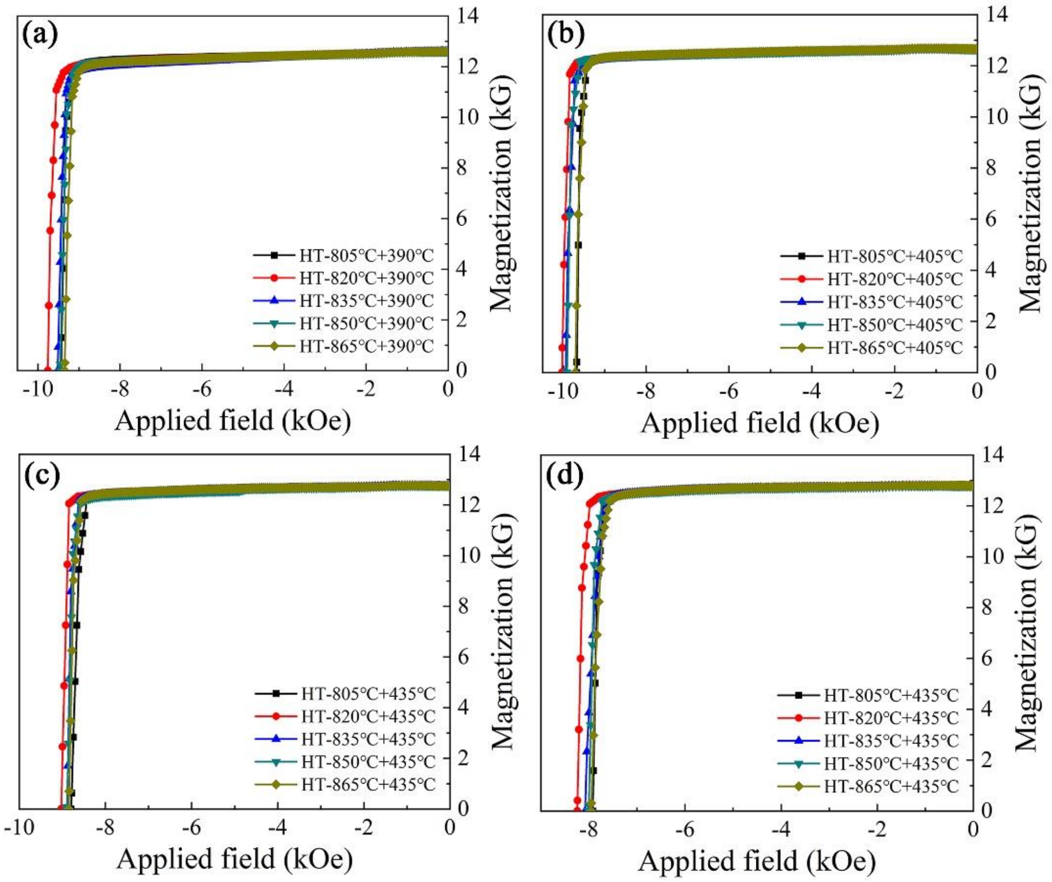

Figure S3. Room-temperature demagnetization curves of (La_xCe_{1-x})-40 (x = 0–0.3) magnets at different annealing temperatures, (a)-(d) corresponding to x = 0–0.3, respectively.

Table S3. The magnetic properties of the (La_xCe_{1-x})-40 (x = 0–0.3) magnets at different annealing temperatures.

La content (x)	Temperatures (°C)	B_r (kG)	H_{cj} (kOe)	$(BH)_{max}$ (MGOe)	H_k/H_{cj} (%)
0	805°C+390°C	12.58	9.44	38.37	97.6
	820°C+390°C	12.58	9.76	38.13	97.2
	835°C+390°C	12.61	9.52	37.23	97.1
	850°C+390°C	12.59	9.46	38.20	97.2
	865°C+390°C	12.60	9.35	37.91	97.2
0.1	805°C+405°C	12.64	9.68	38.81	97.8
	820°C+405°C	12.64	10.03	38.93	98.2
	835°C+405°C	12.63	9.94	38.53	97.8
	850°C+405°C	12.64	9.91	38.91	97.5
	865°C+405°C	12.65	9.71	39.06	97.7
0.2	805°C+435°C	12.75	8.80	39.70	96.4
	820°C+435°C	12.74	9.02	39.34	98.1
	835°C+435°C	12.74	8.89	39.51	97.4
	850°C+435°C	12.75	8.89	39.01	97.2
	865°C+435°C	12.74	8.85	39.59	97.2
0.3	805°C+435°C	12.78	7.92	39.78	97.3
	820°C+435°C	12.78	8.24	39.82	97.2
	835°C+435°C	12.77	8.08	39.83	95.6
	850°C+435°C	12.77	8.00	39.61	97.1
	865°C+435°C	12.79	7.95	39.85	96.1

According to the magnetic property test results in Tables S2 and S3, the optimal two-stage annealing temperatures of (La_xCe_{1-x})-40 (x=0, 0.1, 0.2 and 0.3) magnets are 820°C+390°C, 820°C+405°C, 820°C+435°C and 820°C+435°C, respectively.

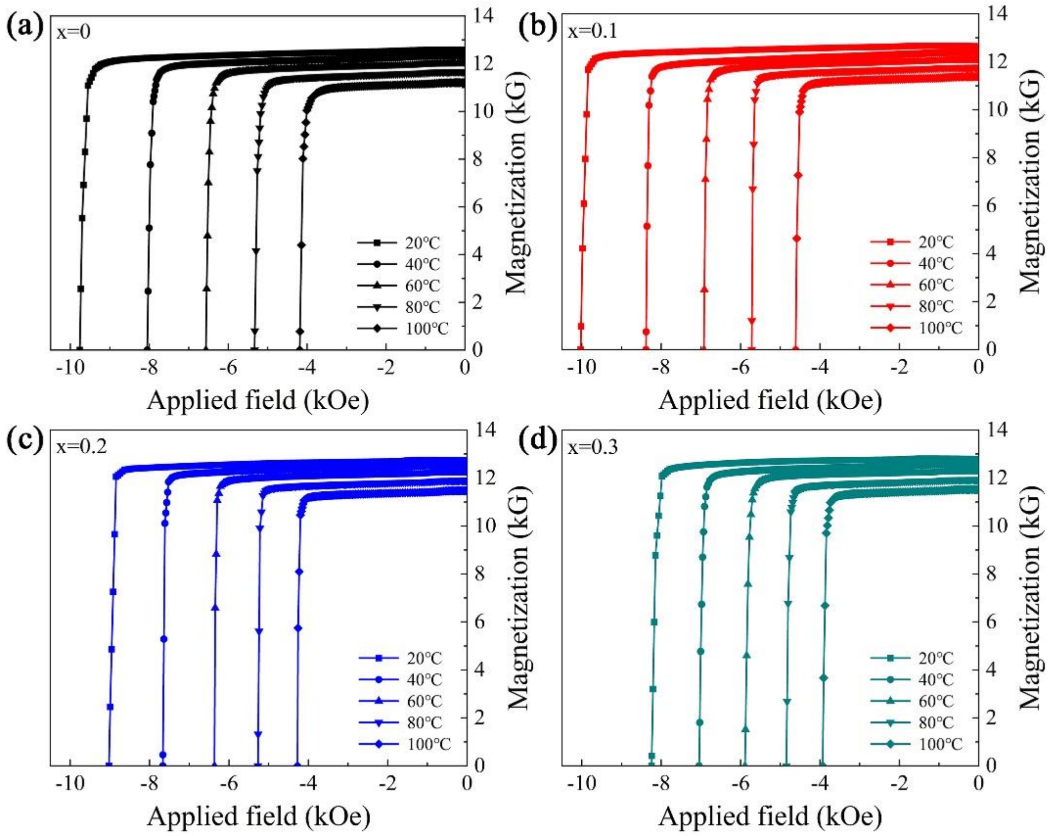

Figure S4. Demagnetization curves at specific temperatures from 20°C to 100°C for (La_xCe_{1-x})-40 (x = 0–0.3) magnets, (a)-(d) corresponding to x = 0–0.3, respectively.

Table S4. The magnetic properties of the (La_xCe_{1-x})-40 (x = 0–0.3) magnets.

La content (x)	Temperatures (°C)	B_r (kG)	H_{cj} (kOe)	$(BH)_{max}$ (MGOe)	H_k/H_{cj} (%)	α (%/°C)	β (%/°C)
0	20	12.58	9.76	38.13	97.2	-	-
	40	12.32	8.05	36.27	97.3	-0.103	-0.876
	60	11.99	6.57	34.02	97.0	-0.117	-0.817
	80	11.67	5.33	31.10	96.0	-0.121	-0.757
	100	11.20	4.19	26.31	95.4	-0.137	-0.713
0.1	20	12.64	10.03	38.93	98.2	-	-
	40	12.40	8.38	36.32	98.4	-0.095	-0.823
	60	12.09	6.92	34.53	98.1	-0.109	-0.775
	80	11.77	5.72	32.48	98.3	-0.115	-0.716
	100	11.38	4.61	28.85	97.1	-0.125	-0.676
0.2	20	12.74	9.02	39.34	98.1	-	-
	40	12.53	7.67	37.60	98.5	-0.082	-0.748
	60	12.22	6.37	35.48	98.9	-0.102	-0.735
	80	11.90	5.27	32.73	98.2	-0.110	-0.693
	100	11.47	4.27	28.74	98.3	-0.124	-0.658
0.3	20	12.78	8.24	39.82	97.2	-	-
	40	12.58	7.04	37.61	97.5	-0.078	-0.728
	60	12.26	5.89	35.34	97.0	-0.102	-0.713
	80	11.94	4.85	31.81	97.3	-0.110	-0.686
	100	11.52	3.93	27.43	96.3	-0.123	-0.654

Table S5. Atomic contents (at. %) of Nd/Pr/Ce/Fe/La elements at the corresponding positions in Figure 4 detected by WDS.

La content (x)	Region	Fe (at. %)	Ce (at. %)	Nd (at. %)	Pr (at. %)	La (at. %)
x=0	1	67.22	26.10	4.75	1.93	-
	2	17.70	28.07	32.05	22.18	-
x=0.1	3	66.87	24.61	5.26	2.10	1.16
	4	13.85	28.55	32.17	19.21	6.22
x=0.2	5	66.18	23.67	5.38	2.43	2.34
	6	14.31	15.36	27.44	20.16	22.73
x=0.3	7	27.35	11.91	20.98	13.25	26.51

Table S6. Atomic contents (at. %) of La/Ce/Pr/Nd elements in 2:14:1 phase of (La_xCe_{1-x})-40 (x = 0–0.3) magnets detected by WDS.

La content (x)	La (at. %)	Ce (at. %)	Pr (at. %)	Nd (at. %)	Fe (at. %)
x=0	-	5.51	2.83	5.54	86.12
x=0.1	0.36	5.10	2.85	5.57	86.12
x=0.2	1.00	4.47	2.87	5.59	86.07
x=0.3	1.57	3.93	2.83	5.61	86.06

Table S7. Atomic contents (at. %) of La/Ce/Pr/Nd/Fe/Cu/Ga elements at the TJ1 and TJ2 positions in Figure 5 detected by EDS.

Positions	La (at. %)	Ce (at. %)	Pr (at. %)	Nd (at. %)	Fe (at. %)	Cu (at. %)	Ga (at. %)
TJ1	-	23.67	2.43	4.55	68.24	0.55	0.56
TJ2	0.98	21.06	2.76	6.80	65.39	1.64	1.37

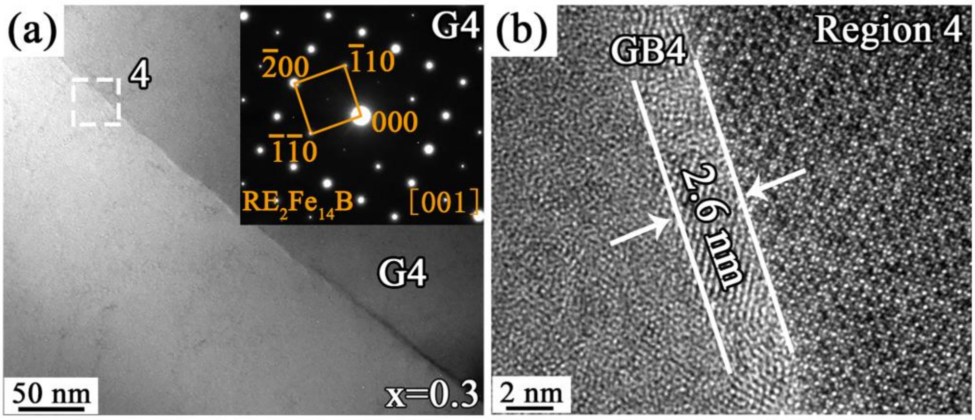

Figure S5. (a) BFI of x = 0.3 magnet, (b) HRTEM images of corresponding GB positions.

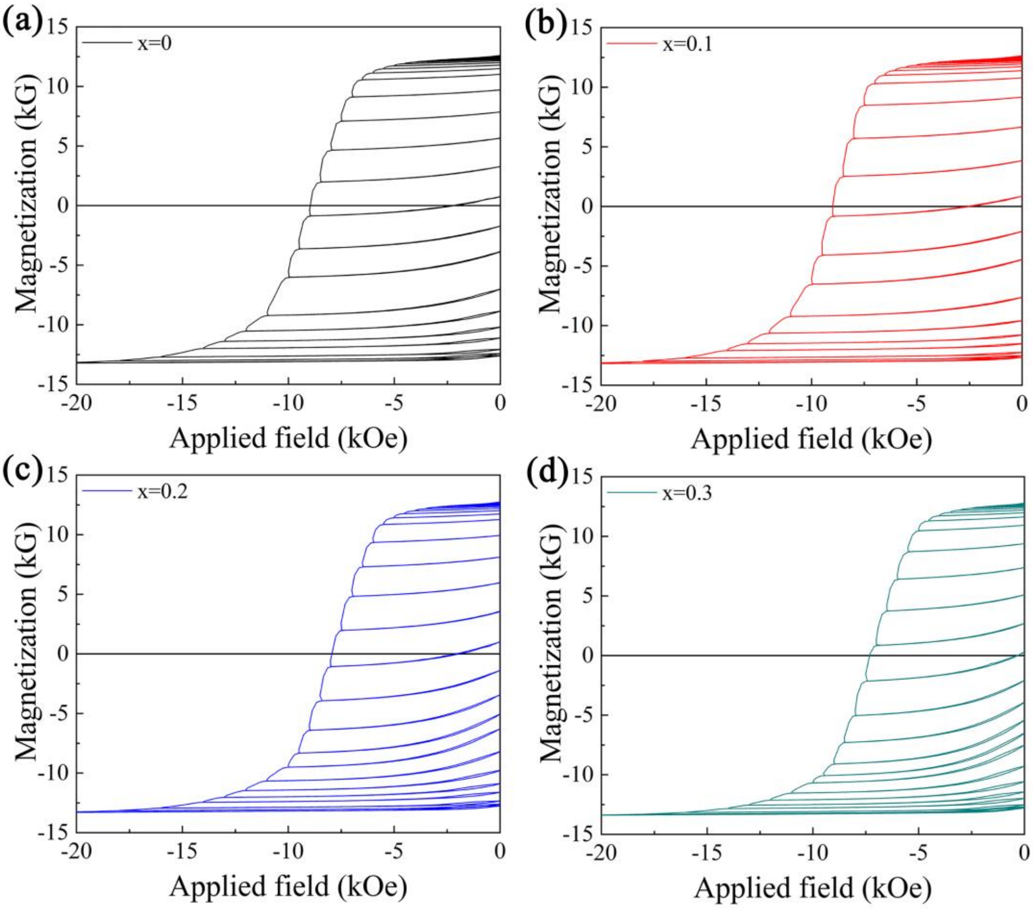

Figure S6. The recoil loops during the magnetization reversal process of (La_xCe_{1-x})-40 (x = 0–0.3) magnets, (a)-(d) corresponding to x = 0–0.3, respectively.

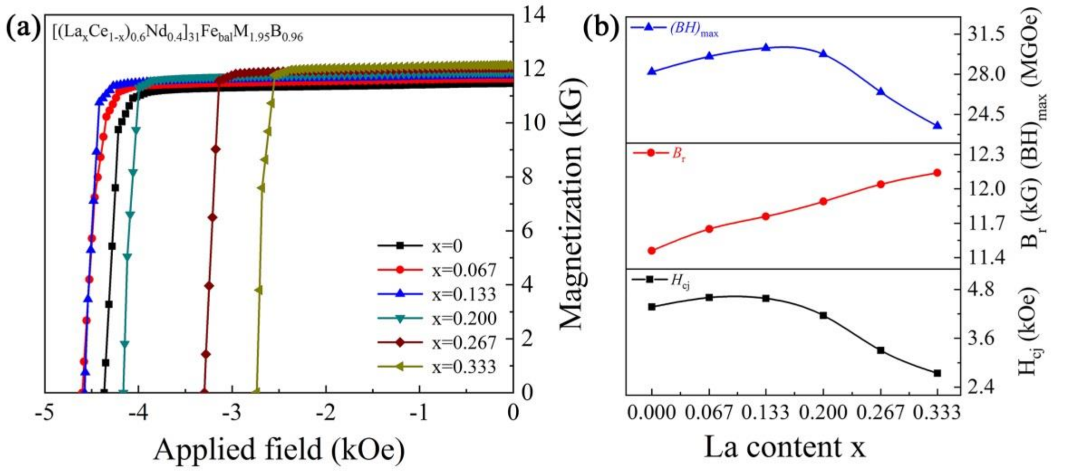

Figure S7. (a) Room-temperature demagnetization curves of $[(La_xCe_{1-x})_{0.6}Nd_{0.4}]_{31}Fe_{bal}M_{1.95}B_{0.96}$ magnets, (b) the variation curves of H_{cj}, B_r and $(BH)_{max}$ with the increase of La substitution.
